# Supplementary material for: Airqtl dissects cell state-specific causal gene regulatory networks with efficient single-cell eQTL mapping
Source: Nat Commun. 2025 Dec 10;16:11403. doi: 10.1038/s41467-025-66214-9 (PMC12739144; doi:10.1038/s41467-025-66214-9)
Supplement: Supplementary file 1 — Supplementary Information [file 41467_2025_66214_MOESM1_ESM.pdf]

# Supplementary Note for "Airqtl dissects cell state-specific causal gene regulatory networks with efficient single-cell eQTL mapping"

## 1 Array of Interleaved Repeats

### 1.1 Definition

Consider $D$ dimensional tensor $\mathbf{X} \in \mathbb{R}^{n_1 \times n_2 \times \cdots \times n_D}$. Define the indexing operator for tensor $\mathbf{X}$ as $\mathbf{X}[(i,j)] \in \mathbb{R}^{n_1 \times n_2 \times \cdots \times n_{i-1} \times n_{i+1} \times \cdots \times n_D}$ as selecting the $j$-th entry of the $i$-th dimension of $\mathbf{X}$, where $i \in \{1,\ldots,D\}$ and $j \in \{1,\ldots,n_i\}$.

Then, we say $\mathbf{X}$ is an Array of Interleaved Repeats (AIR) with $\mathbf{r} \in \mathbb{N}_+^s$ in dimension $d = 1,\ldots,D$ or $\mathbf{X} \in \mathbb{AIR}(d,\mathbf{r})$, subjecting to $\sum_{i=1}^s r_i = n_d$, iff $\forall i \in \{1,\ldots,s\}, j \in \{c_i, c_i+1,\ldots,c_{i+1}-1\}, X[(d,j)] = X[(d,c_i)]$. Here $\mathbf{c} \equiv (c_1, c_2, \ldots, c_{s+1}) \in \mathbb{N}_+^{s+1}$, where $c_1 = 1$, $(c_2,\ldots,c_{s+1}) = c_1 + \operatorname{cumsum}\mathbf{r}$, and $\operatorname{cumsum}$ is the cumulative sum.

More generally, we say $\mathbf{X} \in \mathbb{AIR}(d_1,\mathbf{r}_1; d_2,\mathbf{r}_2;\ldots)$ iff $\forall i, \mathbf{X} \in \mathbb{AIR}(d_i,\mathbf{r}_i)$. Here we introduce additional subscripts to each corresponding variable, such as $d, \mathbf{r}, s, \mathbf{c}$. For simplicity, we say $\mathbf{X} \in \mathbb{AIR}(d_1, d_2, \ldots, \mathbf{r})$ iff $\mathbf{X} \in \mathbb{AIR}(d_1,\mathbf{r}; d_2,\mathbf{r};\ldots)$.

### 1.2 Implementation

AIR is implemented as a compressed data structure for torch.Tensor. For $\mathbf{X} \in \mathbb{AIR}(d_1,\mathbf{r}_1; d_2,\mathbf{r}_2;\ldots)$ where $d_1 > d_2 > \ldots$, its AIR includes the following elements:

- $\widetilde{\mathbf{X}}$: compressed $D$ dimensional data as torch.Tensor, where $\forall i_1 \in \{1,\ldots,s_1\}, i_2 \in \{1,\ldots,s_2\},\ldots$ for each $s_j$ defined as $s$ for the corresponding $i_j$ as $i$ in **Section 1.1**, $\widetilde{\mathbf{X}}[(d_1,i_1)][(d_2,i_2)]\cdots = \mathbf{X}[(d_1,c_{1,i_1})][(d_2,c_{2,i_2})]\ldots$
- $\mathbf{d} = (d_1, d_2, \ldots)$: the dimensions of $\widetilde{\mathbf{X}}$ containing repeats. Each $d_i \in \mathbb{N}_+$.
- $\mathbf{r} = (\mathbf{r}_1, \mathbf{r}_2, \ldots)$: the numbers of repeats for each value in $\widetilde{\mathbf{X}}$. Each $\mathbf{r}_i \in \mathbb{N}_+^{s_i}$.
- $\mathbf{c} = (\mathbf{c}_1, \mathbf{c}_2, \ldots)$: the cumulative sum precomputed from each $\mathbf{r}_i$. Each $\mathbf{c}_i \in \mathbb{N}_+^{s_i+1}$.

### 1.3 Acceleration in matrix operations

Here we provide several non-exhaustive examples of how AIR can accelerate basic matrix operations. Linear mixed models used for sceQTL mapping employ addition, multiplication, and singular value decomposition

Supplementary Table 1: Time complexity comparison of computing addition $\mathbf{X}+\mathbf{Y}$ depending on whether AIR-based acceleration is applied. Here $\mathbf{r} \in \mathbb{N}_+^s$, $s < n_1$, and $s < n_2$.

| $\mathbf{X}$ | $\mathbf{Y}$ | $\mathbf{X}+\mathbf{Y}$ | Without AIR | With AIR |
|---|---|---|---|---|
| $\mathbb{R}^{n_1 \times n_2}$ | $\mathbb{R}^{n_1 \times n_2}$ | $\mathbb{R}^{n_1 \times n_2}$ | $\mathcal{O}(n_1 n_2)$ | $\mathcal{O}(n_1 n_2)$ |
| $\mathbb{R}^{n_1 \times n_2} \cap \mathbb{AIR}(1, \mathbf{r})$ | $\mathbb{R}^{n_1 \times n_2}$ | $\mathbb{R}^{n_1 \times n_2}$ | $\mathcal{O}(n_1 n_2)$ | $\mathcal{O}(n_1 n_2)$ |
| $\mathbb{R}^{n_1 \times n_2} \cap \mathbb{AIR}(1, \mathbf{r})$ | $\mathbb{R}^{n_1 \times n_2} \cap \mathbb{AIR}(1, \mathbf{r})$ | $\mathbb{R}^{n_1 \times n_2} \cap \mathbb{AIR}(1, \mathbf{r})$ | $\mathcal{O}(n_1 n_2)$ | $\mathcal{O}(s n_2)$ |
| $\mathbb{R}^{n_1 \times n_2}$ | $\mathbb{R}$ | $\mathbb{R}^{n_1 \times n_2}$ | $\mathcal{O}(n_1 n_2)$ | $\mathcal{O}(n_1 n_2)$ |
| $\mathbb{R}^{n_1 \times n_2} \cap \mathbb{AIR}(1, \mathbf{r})$ | $\mathbb{R}$ | $\mathbb{R}^{n_1 \times n_2} \cap \mathbb{AIR}(1, \mathbf{r})$ | $\mathcal{O}(n_1 n_2)$ | $\mathcal{O}(s n_2)$ |

Supplementary Table 2: Time complexity comparison of computing multiplication $\mathbf{XY}$ depending on whether AIR-based acceleration is applied. Here $\mathbf{r} \in \mathbb{N}_+^s$, $s < n_X$, and $s < n_3$.

| $\mathbf{X}$ | $\mathbf{Y}$ | $\mathbf{XY}$ | Without AIR | With AIR |
|---|---|---|---|---|
| $\mathbb{R}^{n_X \times n_3}$ | $\mathbb{R}^{n_3 \times n_Y}$ | $\mathbb{R}^{n_X \times n_Y}$ | $\mathcal{O}(n_X n_Y n_3)$ | $\mathcal{O}(n_X n_Y n_3)$ |
| $\mathbb{R}^{n_X \times n_3} \cap \mathbb{AIR}(1, \mathbf{r})$ | $\mathbb{R}^{n_3 \times n_Y}$ | $\mathbb{R}^{n_X \times n_Y} \cap \mathbb{AIR}(1, \mathbf{r})$ | $\mathcal{O}(n_X n_Y n_3)$ | $\mathcal{O}(s n_Y n_3)$ |
| $\mathbb{R}^{n_X \times n_3} \cap \mathbb{AIR}(2, \mathbf{r})$ | $\mathbb{R}^{n_3 \times n_Y}$ | $\mathbb{R}^{n_X \times n_Y}$ | $\mathcal{O}(n_X n_Y n_3)$ | $\mathcal{O}(n_X n_Y s + n_Y n_3)$ |
| $\mathbb{R}^{n_X \times n_3}$ | $\mathbb{R}^{n_3 \times n_Y} \cap \mathbb{AIR}(1, \mathbf{r})$ | $\mathbb{R}^{n_X \times n_Y}$ | $\mathcal{O}(n_X n_Y n_3)$ | $\mathcal{O}(n_X n_Y s + n_X n_3)$ |
| $\mathbb{R}^{n_X \times n_3} \cap \mathbb{AIR}(2, \mathbf{r})$ | $\mathbb{R}^{n_3 \times n_Y} \cap \mathbb{AIR}(1, \mathbf{r})$ | $\mathbb{R}^{n_X \times n_Y}$ | $\mathcal{O}(n_X n_Y n_3)$ | $\mathcal{O}(n_X n_Y s)$ |
| $\mathbb{R}^{n_X \times n_3} \cap \mathbb{AIR}(1, \mathbf{r})$ | $\mathbb{R}$ | $\mathbb{R}^{n_X \times n_3} \cap \mathbb{AIR}(1, \mathbf{r})$ | $\mathcal{O}(n_X n_3)$ | $\mathcal{O}(s n_3)$ |

(SVD) on the genotype matrix or its self-product. Therefore, we focus on these operations in the following examples. For simplicity, we assume $\mathbf{X} \in \mathbb{AIR}(1, \mathbf{r})$ and $\mathbf{Y} \in \mathbb{AIR}(2, \mathbf{r})$.

### 1.3.1 Addition

For AIR, because addition can be operated directly on the compressed data, the time complexity can be improved if the other operand has the same AIR structure or is a scalar (**Tab. 1**).

### 1.3.2 Multiplication

In addition to AIR's acceleration of addition, multiplication also benefits from AIR when the other operand is not an AIR. For example, when $\mathbf{X} \in \mathbb{R}^{n_X \times n_3} \cap \mathbb{AIR}(2, \mathbf{r})$ and $\mathbf{Y} \in \mathbb{R}^{n_3 \times n_Y}$, it is obvious that $\mathbf{XY} = \widetilde{\mathbf{X}}\mathbf{Y}^{(\mathsf{R})}$, where each row of $\mathbf{Y}^{(\mathsf{R})} \in \mathbb{R}^{s \times n_Y}$ is $\mathbf{Y}_i^{(\mathsf{R})} = \sum_{j=c_i}^{c_{i+1}-1} \mathbf{Y}_j$. In this case, the time complexity would be improved to $\mathcal{O}(n_X n_Y s + n_Y n_3)$ from $\mathcal{O}(n_X n_Y n_3)$. Potential accelerations in other multiplication scenarios are shown in **Tab. 2**.

### 1.3.3 Singular value decomposition

Consider matrix $\mathbf{X} \in \mathbb{R}^{n_1 \times n_2} \cap \mathbb{AIR}(1, \mathbf{r}_1; 2, \mathbf{r}_2)$. In other words,

$$\mathbf{X} = \begin{pmatrix} \tilde{x}_{1,1}\mathbf{1}_{r_{1,1}\times r_{2,1}} & \tilde{x}_{1,2}\mathbf{1}_{r_{1,1}\times r_{2,2}} & \cdots & \tilde{x}_{1,s_2}\mathbf{1}_{r_{1,1}\times r_{2,s_2}} \\ \tilde{x}_{2,1}\mathbf{1}_{r_{1,2}\times r_{2,1}} & \tilde{x}_{2,2}\mathbf{1}_{r_{1,2}\times r_{2,2}} & \cdots & \tilde{x}_{2,s_2}\mathbf{1}_{r_{1,2}\times r_{2,s_2}} \\ \cdots & \cdots & \cdots & \cdots \\ \tilde{x}_{s_1,1}\mathbf{1}_{r_{1,s_1}\times r_{2,1}} & \tilde{x}_{s_1,2}\mathbf{1}_{r_{1,s_1}\times r_{2,2}} & \cdots & \tilde{x}_{s_1,s_2}\mathbf{1}_{r_{1,s_1}\times r_{2,s_2}} \end{pmatrix}, \tag{7}$$

where $\mathbf{1}_{i\times j}$ is a $i \times j$ matrix with all entries equal to 1. Its SVD can be performed as:

$$\mathbf{X} = \mathbf{U}\mathbf{\Lambda}\mathbf{V}^*, \tag{8}$$

where $\mathbf{\Lambda}$ is a rectangular diagonal matrix with diagonal elements $(\lambda_1, \lambda_2, \ldots, \lambda_{\min(n_1,n_2)})$. This standard approach has time complexity $\mathcal{O}(n_1 n_2 \min(n_1, n_2))$.

Acceleration of SVD arises from the duplicate rows and columns in $\mathbf{X}$ due to its repeats. First, $\mathbf{X}$ only has (up to) $\min(s_1, s_2)$ nonzero eigenvalues. This allows a low-rank SVD but that would only reduce the time complexity to $\mathcal{O}(n_1 n_2 \min(s_1, s_2))$.

Additionally, we can perform an equivalent eigendecomposition on the rescaled compressed data that accounts for duplicate rows and columns:

$$\tilde{\tilde{\mathbf{X}}} = \left(\tilde{\tilde{x}}_{i,j}\right) \equiv \left(\sqrt{r_{1,i} r_{2,j}}\, \tilde{x}_{i,j}\right) = \tilde{\tilde{\mathbf{U}}}\tilde{\tilde{\mathbf{\Lambda}}}\tilde{\tilde{\mathbf{V}}}^*, \tag{9}$$

where similarly, $\tilde{\tilde{\mathbf{\Lambda}}} = \mathrm{diag}(\tilde{\tilde{\lambda}}_1, \tilde{\tilde{\lambda}}_2, \ldots, \tilde{\tilde{\lambda}}_{\min(s_1,s_2)})$, $\tilde{\tilde{\mathbf{U}}} \equiv (\tilde{\tilde{u}}_{i,j})$, $\tilde{\tilde{\mathbf{V}}} \equiv (\tilde{\tilde{v}}_{i,j})$, and $^*$ means conjugate transpose. It is easy to see that $\mathbf{\Lambda} = \tilde{\tilde{\mathbf{\Lambda}}}$ (for nonzero entries). In addition, $\mathbf{U} \in \mathbb{R}^{n_1 \times \min(s_1,s_2)} \cap \mathbb{AIR}(1, \mathbf{r}_1)$ has its compressed data $\widetilde{\mathbf{U}} \equiv (\tilde{u}_{i,j}) = (\tilde{\tilde{u}}_{i,j}/\sqrt{r_{1,i}})$ and $\mathbf{V} \in \mathbb{R}^{n_2 \times \min(s_1,s_2)} \cap \mathbb{AIR}(1, \mathbf{r}_2)$ has its compressed data $\widetilde{\mathbf{V}} \equiv (\tilde{v}_{i,j}) = (\tilde{\tilde{v}}_{i,j}/\sqrt{r_{2,i}})$. Therefore, AIR acceleration allows SVD to be directly performed on compressed data and further reduces its time complexity to $\mathcal{O}(s_1 s_2 \min(s_1, s_2))$.

## 1.4 Application in sceQTL mapping

AIR provides efficient storage and processing of genotype data for population-scale scRNA-seq studies. Given $n_{\text{cell}}$ cells from $n_{\text{donor}}$ donors, with $n_{\text{SNP}}$ SNPs and $n_{\text{gene}}$ genes, the raw genotype matrix $\widetilde{\mathbf{G}} \in \mathbb{R}^{n_{\text{SNP}} \times n_{\text{donor}}}$ and normalized expression matrix $\mathbf{E} \in \mathbb{R}^{n_{\text{gene}} \times n_{\text{cell}}}$ form the core inputs. The genotype matrix requires expansion to cell level for analysis, represented as $\mathbf{G}^{(E)} \in \mathbb{R}^{n_{\text{SNP}} \times n_{\text{cell}}} \cap \mathbb{AIR}(2, \mathbf{r})$, where $\mathbf{r}$ contains cell counts per donor

$(\sum_i r_i = n_{\text{cell}})$.

Using the algorithms proposed in **Section 1.3**, AIR can accelerate several critical steps in sceQTL mapping:

- **GRM estimation.** The GRM estimated from genotype matrix $\mathbf{K} = \left(\mathbf{G}^{(\mathsf{E})}\right)^T \mathbf{G}^{(\mathsf{E})} \in \mathbb{R}^{n_{\text{cell}} \times n_{\text{cell}}} \cap \mathbb{AIR}(1, 2, \mathbf{r})$, after normalization of $\mathbf{G}^{(\mathsf{E})}$. Because $\mathbf{G}^{(E)} \in \mathbb{R}^{n_{\text{SNP}} \times n_{\text{cell}}} \cap \mathbb{AIR}(2, \mathbf{r})$, AIR reduces its time complexity from $\mathcal{O}(n_{\text{SNP}} n_{\text{cell}}^2)$ to $\mathcal{O}(n_{\text{SNP}} n_{\text{donor}}^2)$.

- **GRM factorization.** The GRM's SVD decomposition $\mathbf{K} = \mathbf{U}\mathbf{\Lambda}\mathbf{U}^T$, where factors $\mathbf{U} \in \mathbb{R}^{n_{\text{donor}} \times n_{\text{cell}}} \cap \mathbb{AIR}(2, \mathbf{r})$. Because $\mathbf{K} \in \mathbb{R}^{n_{\text{cell}} \times n_{\text{cell}}} \cap \mathbb{AIR}(1, 2, \mathbf{r})$, AIR reduces its time complexity from $\mathcal{O}(n_{\text{cell}}^2 n_{\text{donor}})$ to $\mathcal{O}(n_{\text{donor}}^3)$.

- **GRM transformation.** The transformation of genotype and expression matrices by GRM factors, as $\mathbf{G}^{(\mathsf{E})}\mathbf{U}^T \in \mathbb{R}^{n_{\text{SNP}} \times n_{\text{donor}}}$ and $\mathbf{E}\mathbf{U}^T \in \mathbb{R}^{n_{\text{gene}} \times n_{\text{donor}}}$. Because $\mathbf{G}^{(E)} \in \mathbb{R}^{n_{\text{SNP}} \times n_{\text{cell}}} \cap \mathbb{AIR}(2, \mathbf{r})$ and $\mathbf{U} \in \mathbb{R}^{n_{\text{donor}} \times n_{\text{cell}}} \cap \mathbb{AIR}(2, \mathbf{r})$, AIR reduces its time complexity from $\mathcal{O}((n_{\text{SNP}} + n_{\text{gene}}) n_{\text{cell}} n_{\text{donor}})$ to $\mathcal{O}((n_{\text{SNP}} + n_{\text{gene}}) n_{\text{donor}}^2)$.

- **SNP-gene covariance.** The raw covariance of genotype and expression matrices, as $\mathbf{G}^{(\mathsf{E})}\mathbf{E}^T / n_{\text{cell}} \in \mathbb{R}^{n_{\text{SNP}} \times n_{\text{gene}}}$. Because $\mathbf{G}^{(E)} \in \mathbb{R}^{n_{\text{SNP}} \times n_{\text{cell}}} \cap \mathbb{AIR}(2, \mathbf{r})$, AIR reduces its time complexity from $\mathcal{O}(n_{\text{SNP}} n_{\text{gene}} n_{\text{cell}})$ to $\mathcal{O}(n_{\text{SNP}} n_{\text{gene}} n_{\text{donor}})$.

- Other calculations such as covariance of genotype or gene expression matrix with covariates. These calculations are minor and do not determine the time complexity or practical running time.

For a high-level description, also see **Fig. 1a** and “SceQTL mapping — AIR accelerations” in **Methods**.

# 2 Supplementary Results

## 2.1 Comparison of single-cell normalization

We evaluated two normalization approaches within the airqtl framework: Normalisr (default) and log(CPM+10K) (namely LogCPM), assessing their impact on both standard and cell type-specific sceQTL mapping. LogCPM serves as a widely-used baseline method in scRNA-seq normalization studies. Our benchmarking utilized the same datasets and methodology as the airqtl-CellRegMap comparisons.

In standard sceQTL mapping, LogCPM exhibited elevated false positive rates (**Supplementary Figure 1ab**) alongside expression-dependent underestimation bias of effect sizes (**Supplementary Figure 1cd**), mirroring its previously documented limitations in scRNA-seq differential expression analyses [5].

The main text proposed that expression-dependent effect size biases could spuriously inflate cell type-specific signals, where mere expression-level changes might be misinterpreted as cell type-specific regulation. While CellRegMap's computational limitations prevented direct validation, airqtl's framework enabled testing this hypothesis by applying LogCPM normalization with identical acceleration benefits.

Consistent with our hypothesis, LogCPM demonstrated significantly higher false positive rates in cell type-specific sceQTL detection compared to Normalisr (**Supplementary Figure 4a**). Furthermore, LogCPM underperformed Normalisr across all statistical benchmarks (**Supplementary Figure 4bc**), confirming the importance of proper normalization for accurate cell type-specific analyses.

## 3 Supplementary Figures

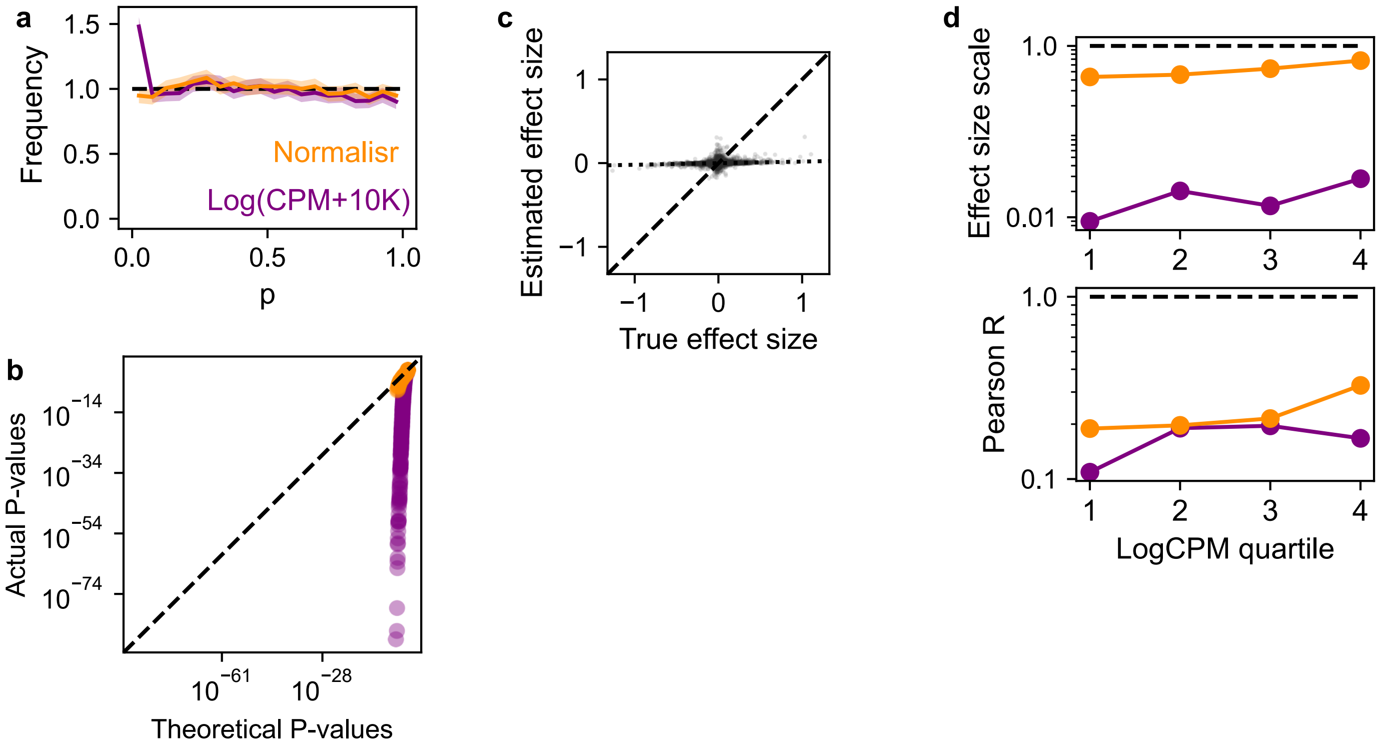


Supplementary Figure 1: **Superior statistical performance of Normalisr versus LogCPM in sceQTL mapping.** **ab** P-value distribution histograms (**a**) and QQ plots (**b**) for non-eQTLs show LogCPM's elevated false positive rate. Error bars in histograms were estimated as $3\sqrt{N}$ where $N$ is the number of entries in each bin. **c** Ground-truth (X) and estimated (Y) sceQTL effect sizes (dots) by LogCPM without stratification. Deviation of the best-fit linear model (dotted line) from the diagonal (dashed line) indicates the extent of overall effect size underestimation. **d** Effect size estimation bias (top) and variance (bottom) for genes across expression quartiles (X). Dashed line: perfect performance. Color: method.

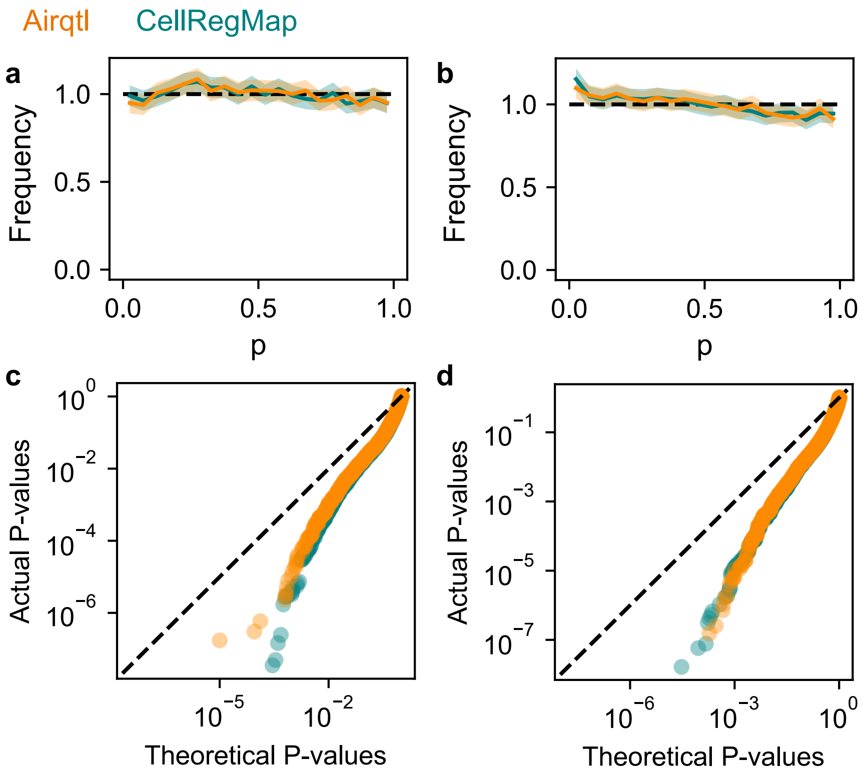


Supplementary Figure 2: **Sensitivity and specificity benchmarking for sceQTL mapping.** P-value distribution histograms (**ab**) and quantile-quantile plots (**cd**) for non-eQTLs (**ac**) and eQTLs (**bd**) as computed by different methods (color) from simulated data. Error bars in histograms were estimated as $3\sqrt{N}$ where $N$ is the number of entries in each bin. Dashed line: perfect performance.

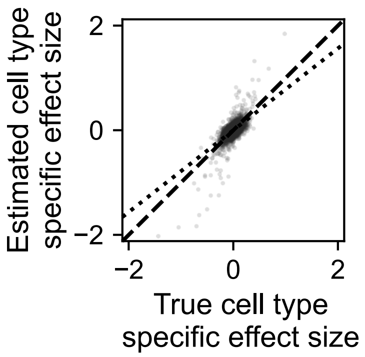


Supplementary Figure 3: **Airqtl provided accurate estimation of cell type-specific eQTL effect sizes.** Scatter plot of estimated (Y) and true (X) eQTL cell type-specific effect sizes as shown by individual SNP-gene pairs (dot) and best linear fit (dotted line). Dashed line: perfect performance with Y=X.

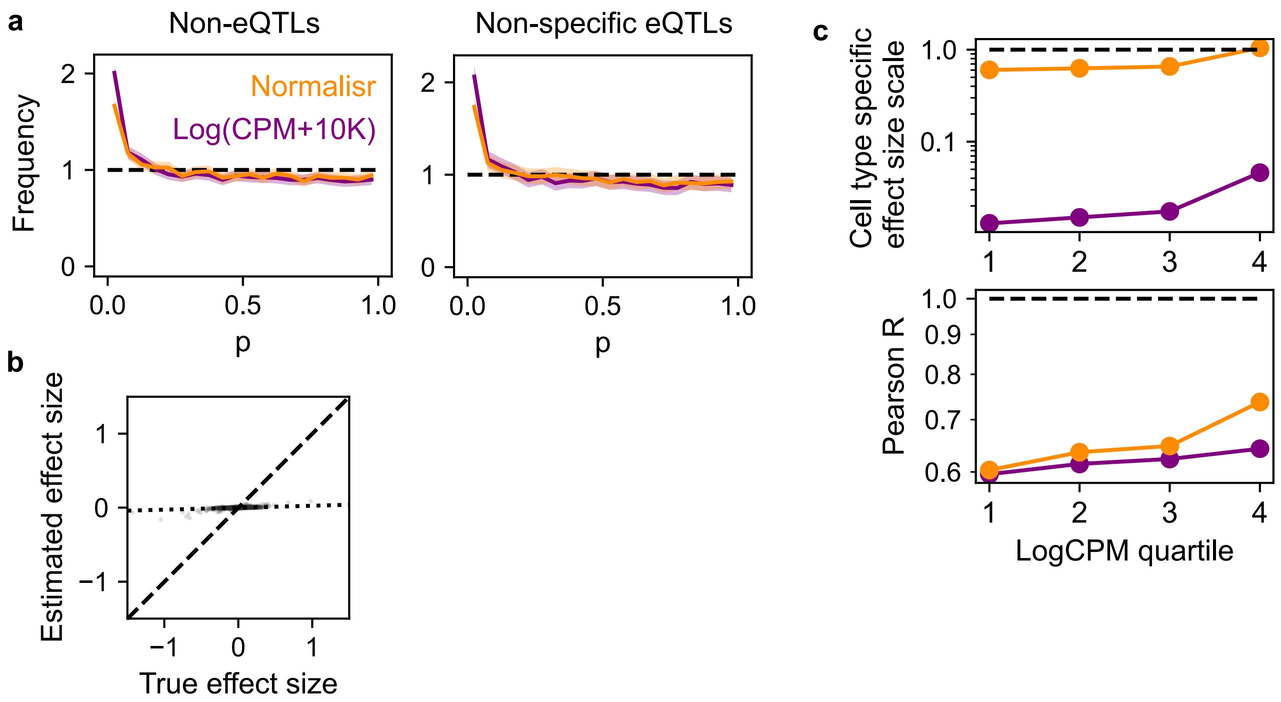


Supplementary Figure 4: **Normalisr outperforms LogCPM in cell type-specific sceQTL mapping.** **a** P-value distribution histograms for non-eQTLs (left) and non-specific eQTLs (right) without calibration show LogCPM's increased false positives. Error bars in histograms were estimated as $3\sqrt{N}$ where $N$ is the number of entries in each bin. **b** Ground-truth (X) and estimated (Y) cell type-specific sceQTL effect sizes (dots) by LogCPM without stratification. Deviation of the best-fit linear model (dotted line) from the diagonal (dashed line) indicates the extent of overall effect size underestimation. **c** Effect size estimation bias (top) and variance (bottom) for genes across expression quartiles (X). Dashed line: perfect performance. Color: method.

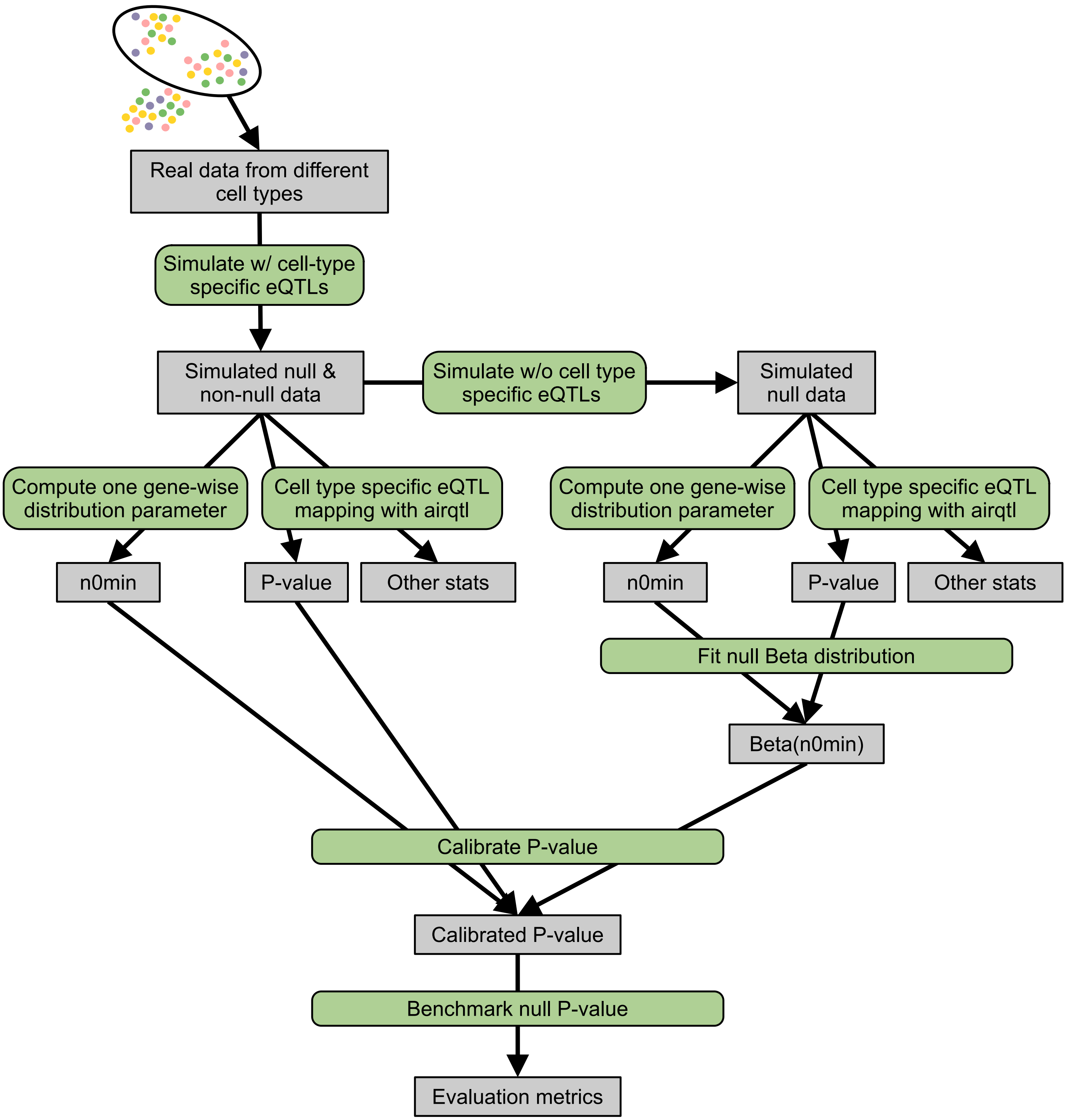


Supplementary Figure 5: **Workflow to objectively optimize parameter choice for P-value calibration.** Each candidate parameter was separately considered with this workflow to produce evaluation metrics. This workflow demonstration uses n0min as an example but other parameters (e.g. lcpm) followed the same workflow and use the same simulated data. Candidate parameter with the best evaluation results was selected for P-value calibration for this dataset (**Methods**).

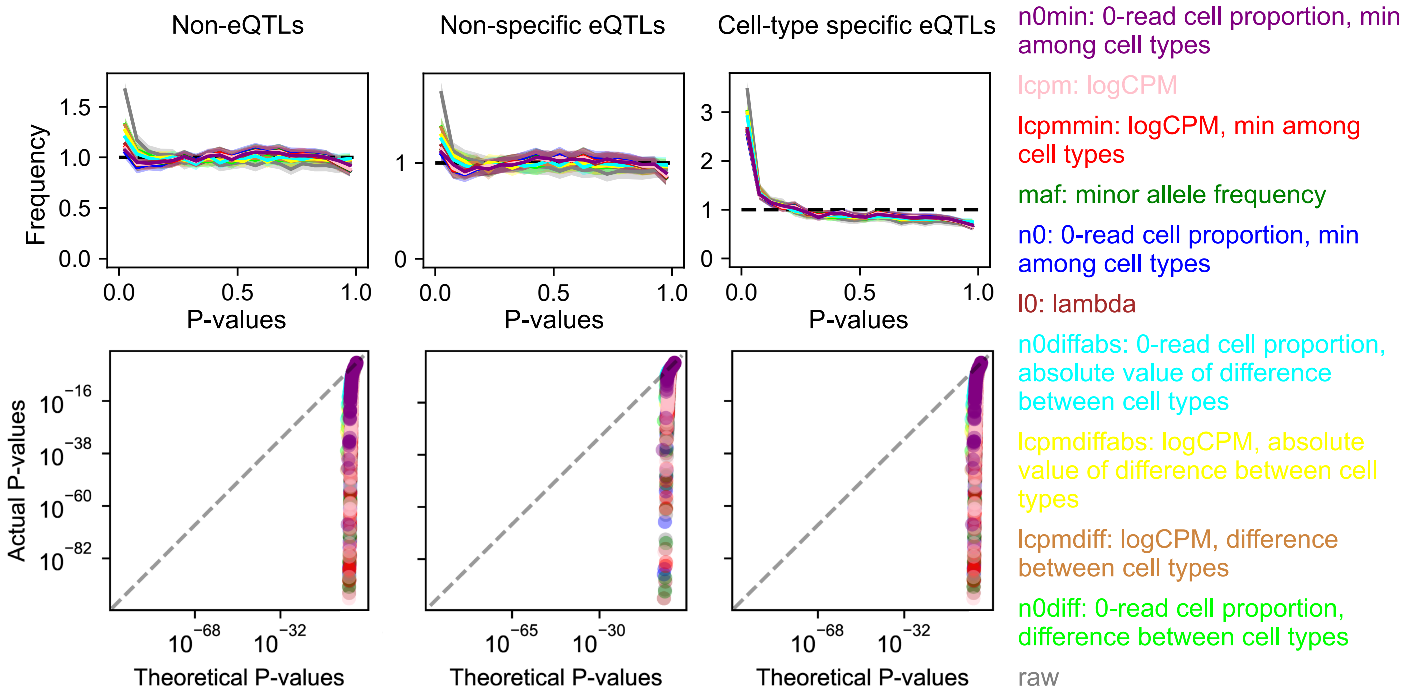


Supplementary Figure 6: **Objective optimization of cell type-specific sceQTL mapping through sensitivity and specificity benchmarking.** P-value distribution histograms (top) and quantile-quantile plots (bottom) for non-eQTLs (left), non-specific eQTLs (center), and cell type-specific eQTLs (right) as calibrated by different models, each with a different calibration parameter (color and legend, ordered by KS test P of non-specific eQTLs against uniform distribution). Raw P values were computed from the vanilla model with no calibration. Error bars in histograms were estimated as $3\sqrt{N}$ where $N$ is the number of entries in each bin. Dashed line: perfect performance (uniform distribution).

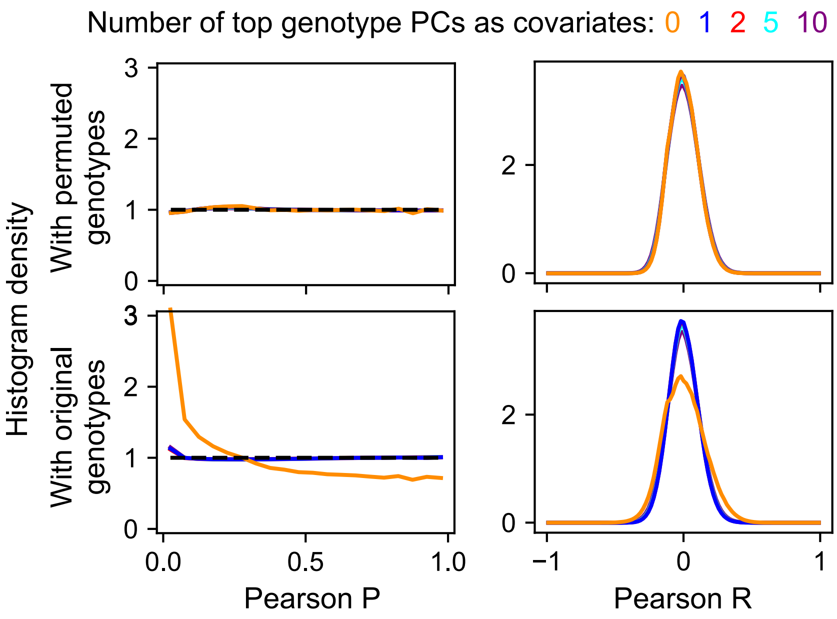


Supplementary Figure 7: **Pearson test for correlation between genotype pairs on different chromosomes reveals the best number of genotype PCs to model their covariance from population structure in Randolph et al dataset.** Pearson P-value (left) and R (right) distribution histograms for genotype pairs on different chromosomes to reflect correlation between them due to population structure and the best parameter to account for them. Tests were performed separately for original genotypes (bottom) and genotypes that were independently randomly permuted between donors (top). Different numbers of principal components computed from all genotypes were used as covariates (color). One genotype PC was the minimum number that provided the best removal of population structure effects.

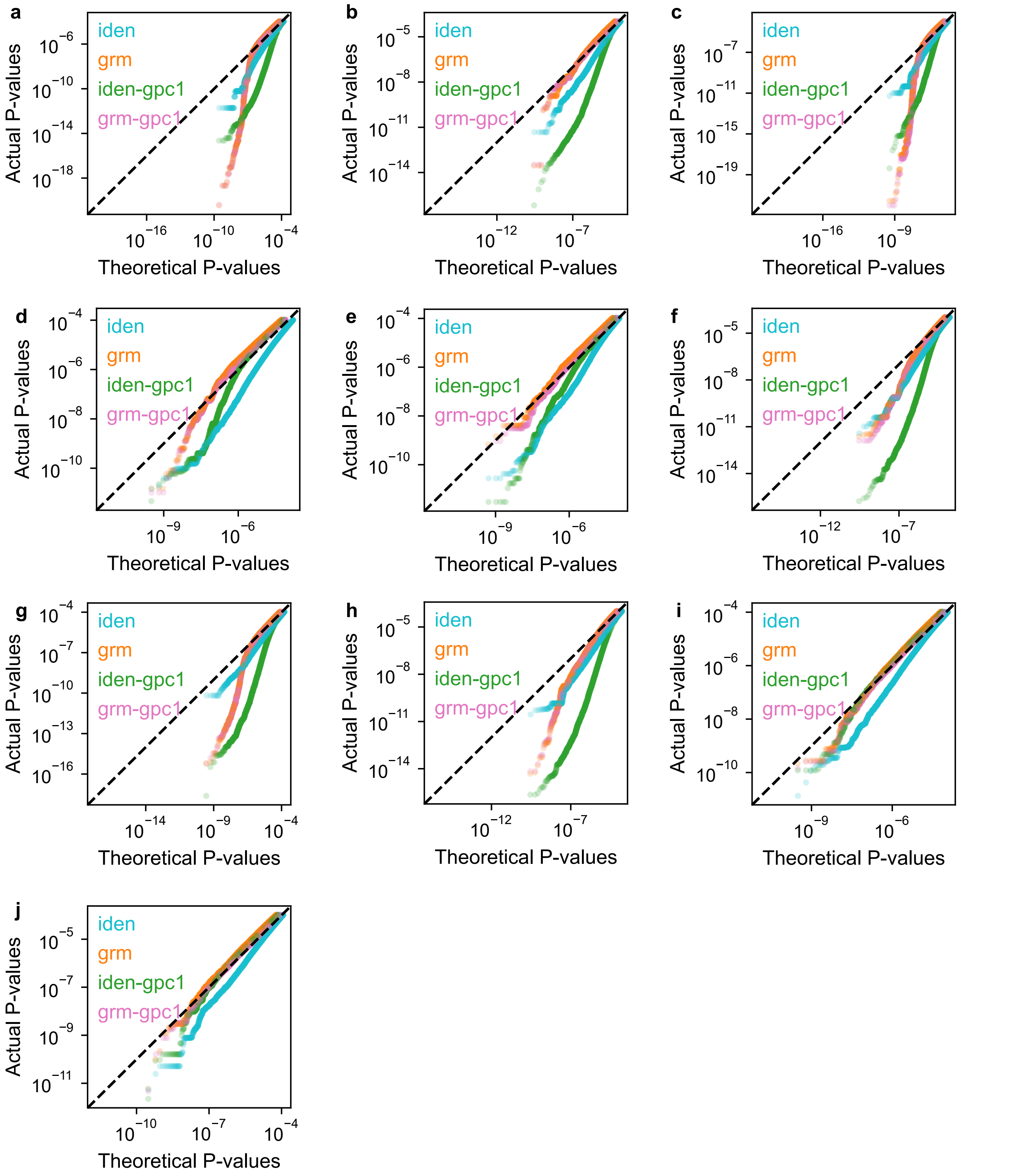


Supplementary Figure 8: **QQ plots of null trans-sceQTL P-values by different methods to accounting for admixed populations.** Panel corresponds to cell states, including B cells, flu (**a**); B cells, NI (**b**); CD8+ T cells, flu (**c**); CD8+ T cells, NI (**d**); highly infected cells, flu (**e**); infected monocytes, flu (**f**); monocytes, flu (**g**); monocytes, NI (**h**); NK cells, flu (**i**); and NK cells, NI (**j**). QQ plots for CD4+ T cells are in **Fig. 5c**. Color indicates method, including identity donor-level GRM (iden), GRM estimated from genotypes (grm), identity donor-level GRM with 1 top genotype PC as covariate (iden-gpc1), and GRM estimated from genotypes with 1 top genotype PC as covariate (grm-gpc1).

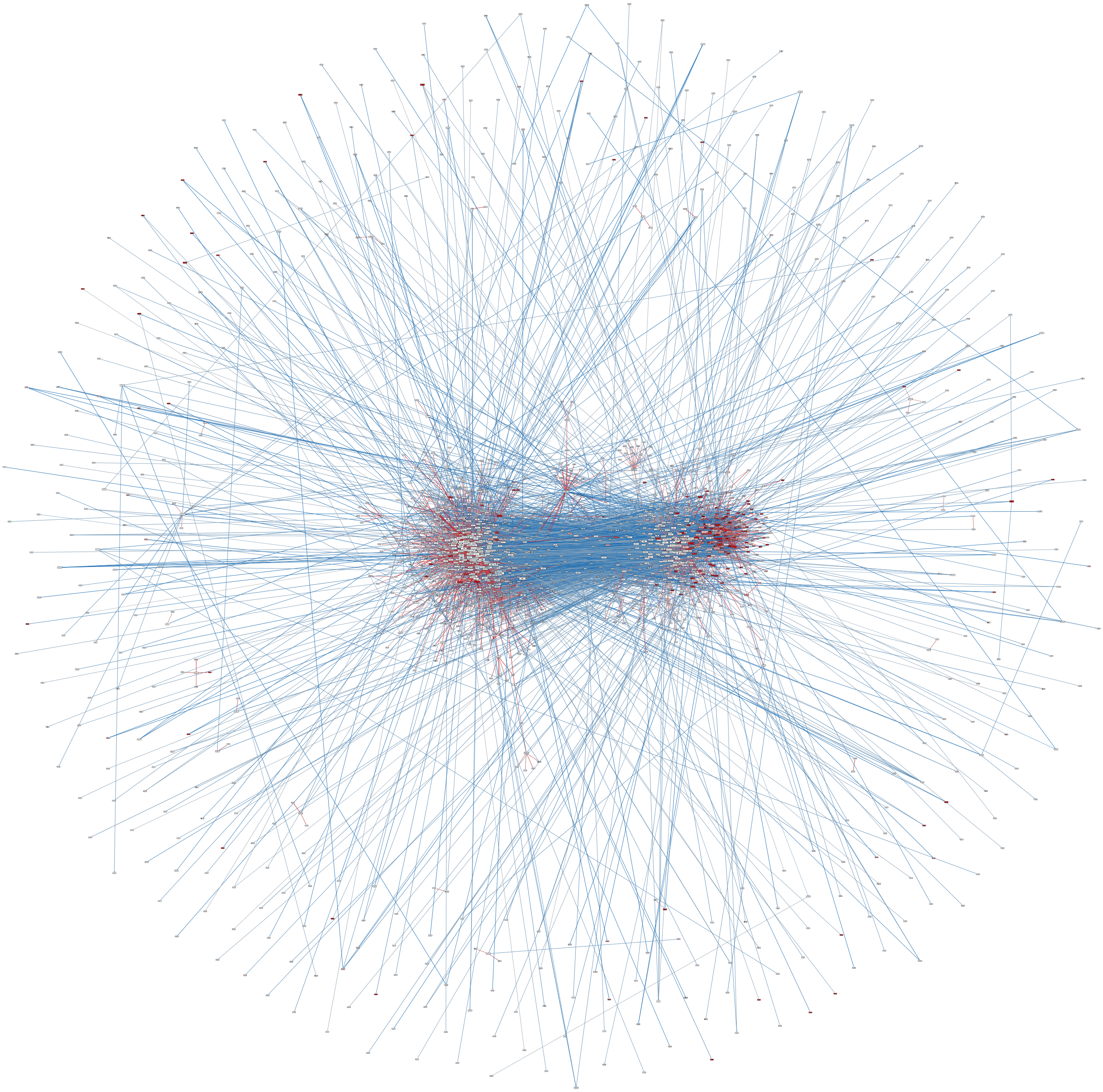

Supplementary Figure 9: **Overview of inferred cGRN for CD4+ T cells under flu condition.** Upregulated genes (red nodes) are enriched in one cluster (right). Magnification is recommended to distinguish red nodes from red edges. Only the largest connected component is shown. Node: gene. Edge: gene regulation. Node color: logFC in expression flu v.s. NI (red: positive; blue: negative; white: zero). Edge color: gene regulation effect size under flu condition (red: positive; blue: negative). See **Supplementary Data 5** for cGRN data.

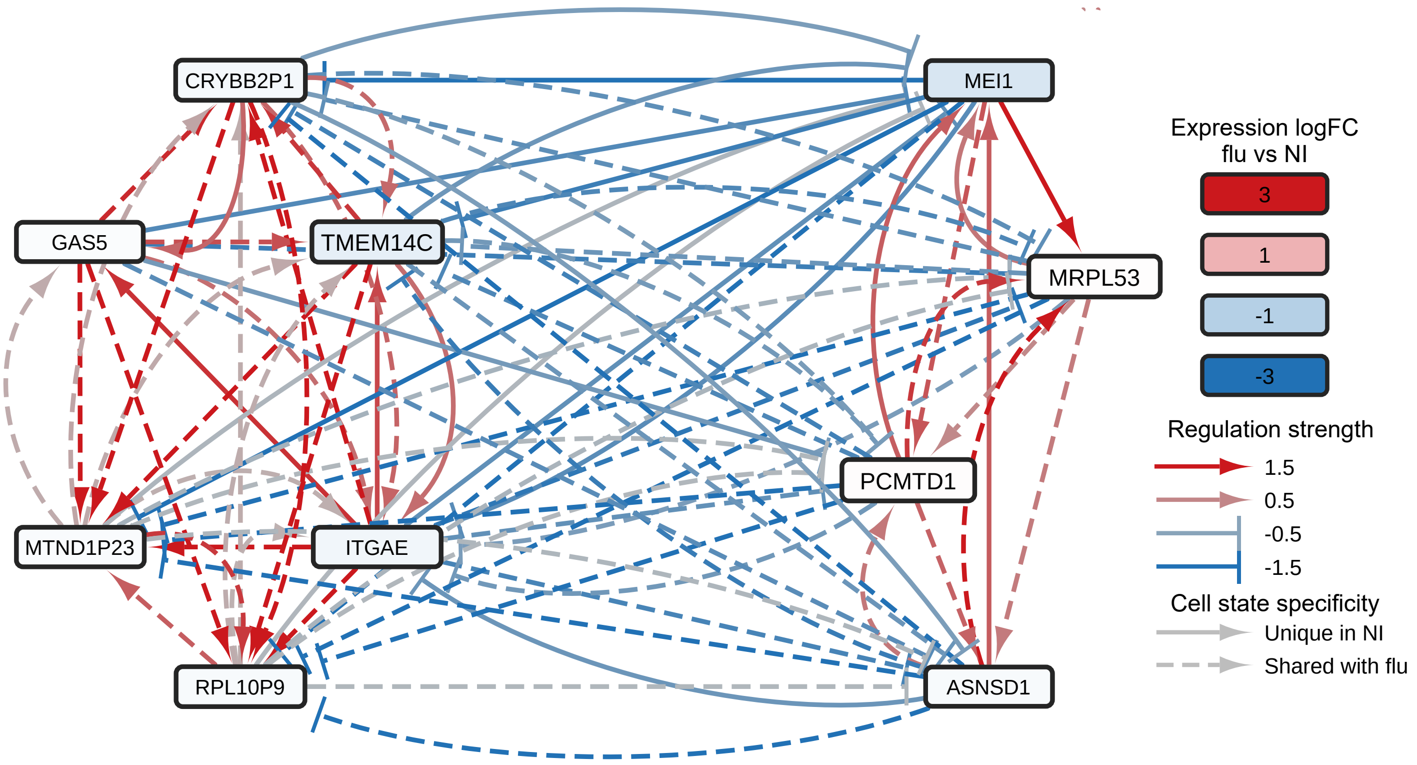


Supplementary Figure 10: **Sub-cGRN between top master regulators in CD4+ T cells under NI condition.**

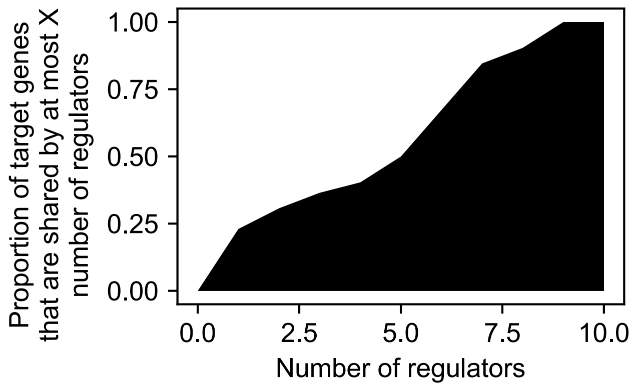


Supplementary Figure 11: **Master regulators had unique target genes not shared with other master regulators.** A large fraction (∼20%, Y) of "defense response to virus" target genes of master regulators were unique to one master regulator in CD4+ T cells under flu condition.

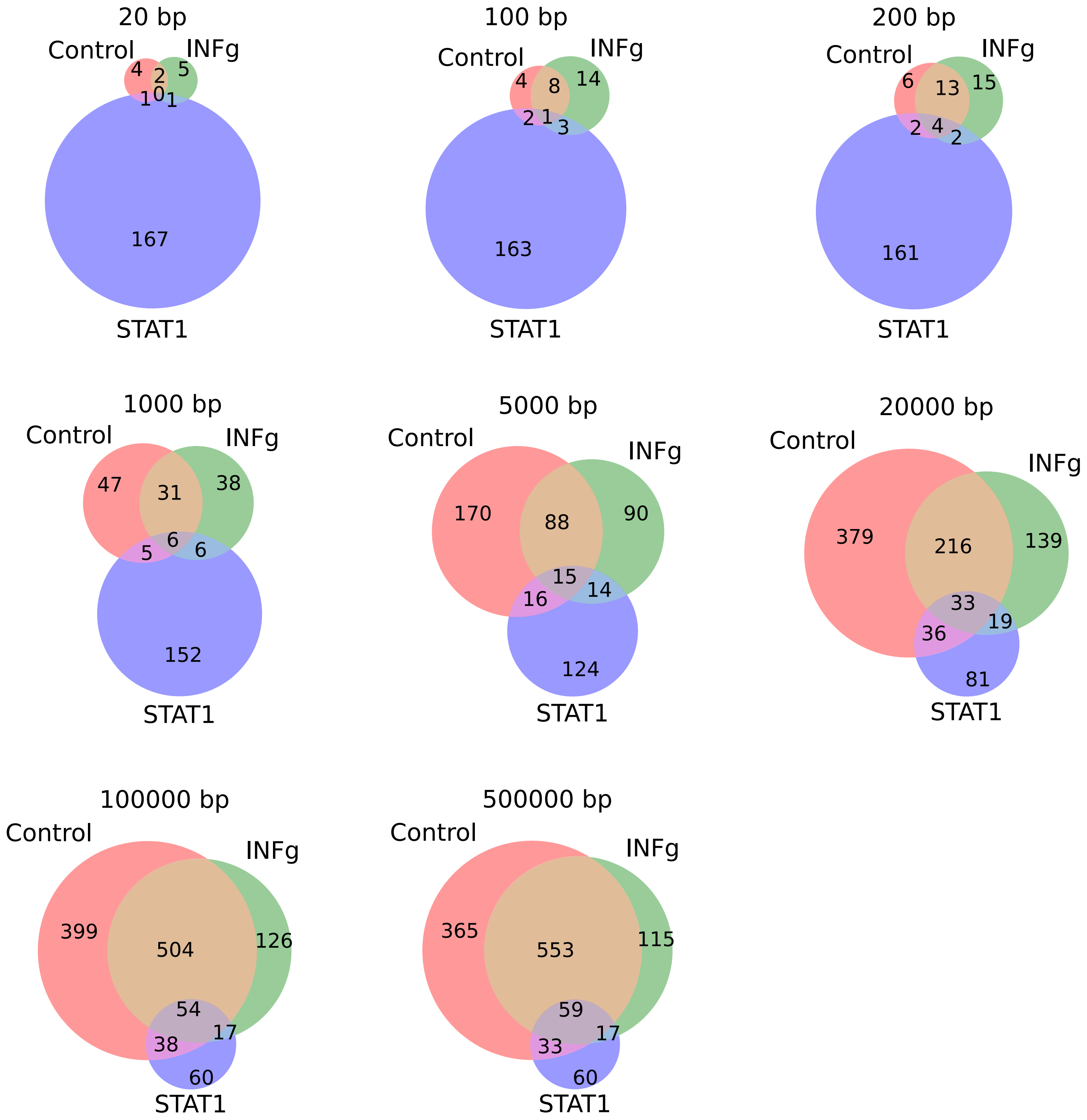


Supplementary Figure 12: **Concordance between airqtl-inferred and ChIP-seq-identified *STAT1* target genes.** Venn diagrams compare airqtl-inferred *STAT1* targets (direct+indirect) with genes near STAT1 binding sites at varying genomic distances (panel title) in human CD4+ T cells, with (INFg) or without (Control) stimulation. The substantial but incomplete overlap confirms airqtl's ability to recover both direct ChIP-seq-detectable targets and indirect ChIP-seq-undetectable targets.

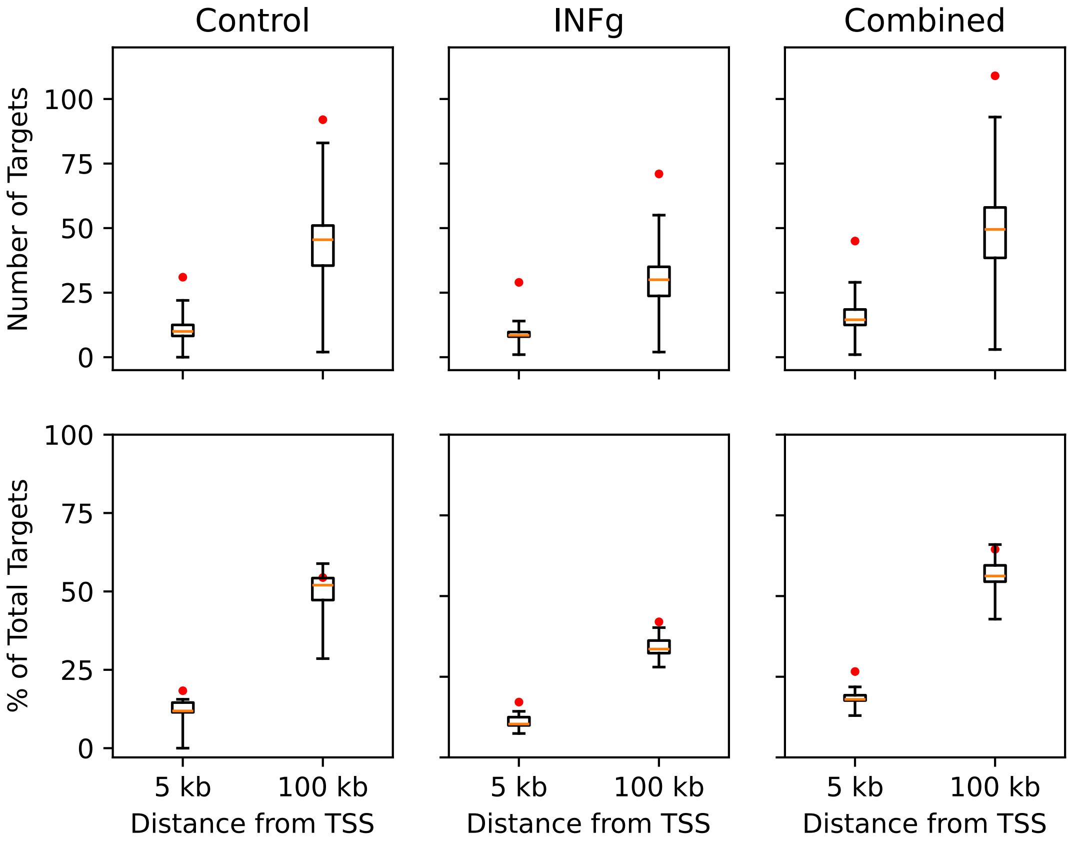


Supplementary Figure 13: **High specificity of airqtl-inferred *STAT1* target genes relative to ChIP-seq data.** Target overlap metrics (Y) between airqtl predictions and ChIP-seq results are shown as: (top) gene counts and (bottom) percentage of predicted targets. Boxplots display baseline distributions for other (n=9) master regulators against STAT1 ChIP-seq results, with orange dots marking *STAT1*-specific results. Analyses compare different genomic distances (X) and conditions (columns), revealing stronger agreement for proximal (v.s. distal) and INFg-stimulated (v.s. Control) targets — consistent with known *STAT1* biology and validating airqtl's cGRN inference accuracy. Combined: *STAT1* targets found in either Control or INFg conditions. Whiskers indicate extrema.
