# Supplementary material for: Airqtl dissects cell state-specific causal gene regulatory networks with efficient single-cell eQTL mapping
Source: Nat Commun. 2025 Dec 10;16:11403. doi: 10.1038/s41467-025-66214-9 (PMC12739144; doi:10.1038/s41467-025-66214-9)
Supplement: Supplementary file 2 — Description of Additional Supplementary Files [file 41467_2025_66214_MOESM2_ESM.pdf]

Description of Additional Supplementary Files

Supplementary Data 1. KS test statistics when comparing each method's sceQTL mapping P values between null and non-null cases or against standard uniform distribution.

Supplementary Data 2. KS test statistics when comparing airqtl's cell type-specific sceQTL mapping P values between null (non-eQTLs and non-specific eQTLs separately) and non-null (specific eQTLs) cases or against standard uniform distribution.

Supplementary Data 3. SceQTL mapping summary statistics for each cell state in the Randolph et al dataset, with cutoffs first on raw P$< 10^{-4}$ for trans-sceQTLs and then BH Q$<$0.1 separately for cis- and trans-sceQTLs.

Supplementary Data 4. Exact P-values in **Fig. 5bc**.

Supplementary Data 5. Node and edge properties of the inferred cell state-specific cGRNs.

Supplementary Data 6. GO enrichment results for the target genes of each master regulator of CD4+ T cells under flu or NI condition, separately for activation and repression targets.

Supplementary Data 7. Hypergeometric test statistics for the overlap between airqtl-inferred *STAT1* targets (direct+indirect) with genes near STAT1 binding sites in human CD4+ T cells.
