# Supplementary material for: Airqtl dissects cell state-specific causal gene regulatory networks with efficient single-cell eQTL mapping
Source: Nat Commun. 2025 Dec 10;16:11403. doi: 10.1038/s41467-025-66214-9 (PMC12739144; doi:10.1038/s41467-025-66214-9)
Supplement: Supplementary file 10 — Reporting Summary [file 41467_2025_66214_MOESM10_ESM.pdf]

nature portfolio

Corresponding author(s): Lingfei Wang
Last updated by author(s): 2025/10/27

Reporting Summary

Nature Portfolio wishes to improve the reproducibility of the work that we publish. This form provides structure for consistency and transparency in reporting. For further information on Nature Portfolio policies, see our Editorial Policies and the Editorial Policy Checklist.

Statistics

For all statistical analyses, confirm that the following items are present in the figure legend, table legend, main text, or Methods section.

n/a	Confirmed	
☐	☒	The exact sample size (n) for each experimental group/condition, given as a discrete number and unit of measurement
☒	☐	A statement on whether measurements were taken from distinct samples or whether the same sample was measured repeatedly
☐	☒	The statistical test(s) used AND whether they are one- or two-sided<br>*Only common tests should be described solely by name; describe more complex techniques in the Methods section.*
☐	☒	A description of all covariates tested
☐	☒	A description of any assumptions or corrections, such as tests of normality and adjustment for multiple comparisons
☐	☒	A full description of the statistical parameters including central tendency (e.g. means) or other basic estimates (e.g. regression coefficient) AND variation (e.g. standard deviation) or associated estimates of uncertainty (e.g. confidence intervals)
☐	☒	For null hypothesis testing, the test statistic (e.g. F, t, r) with confidence intervals, effect sizes, degrees of freedom and P value noted<br>*Give P values as exact values whenever suitable.*
☒	☐	For Bayesian analysis, information on the choice of priors and Markov chain Monte Carlo settings
☒	☐	For hierarchical and complex designs, identification of the appropriate level for tests and full reporting of outcomes
☐	☒	Estimates of effect sizes (e.g. Cohen's d, Pearson's r), indicating how they were calculated

Our web collection on statistics for biologists contains articles on many of the points above.

Software and code

Policy information about availability of computer code

Data collection	Not applicable
Data analysis	Airqtl 0.2.0 (doi: 10.5281/zenodo.14658032), Normalisr 1.0.0, CellRegMap c9da8e212f6e5ec9734538416193e635ccbcd88b, Cytoscape 3.10.2, GOATOOLS 1.3.11, mygene 3.2.2, fdrtool 1.2.18, Homer 5.1

For manuscripts utilizing custom algorithms or software that are central to the research but not yet described in published literature, software must be made available to editors and reviewers. We strongly encourage code deposition in a community repository (e.g. GitHub). See the Nature Portfolio guidelines for submitting code & software for further information.

Data

Policy information about availability of data

All manuscripts must include a data availability statement. This statement should provide the following information, where applicable:

- Accession codes, unique identifiers, or web links for publicly available datasets
- A description of any restrictions on data availability
- For clinical datasets or third party data, please ensure that the statement adheres to our policy

The Randolph et al population-scale scRNA-seq dataset is publicly available on Zenodo under accession code 4273998. Transcription factor and motif list was downloaded from https://resources.aertslab.org/cistarget/motif_collections/v10nr_clust_public/snapshots/motifs-v10-nr.hgnc-m0.00001-o0.0.tbl. The differential expression analysis result of STAT1 perturbation was downloaded from Supplementary Data of [54]. ChIP-seq of STAT1 was downloaded from GEO under accession GSE27158.

Research involving human participants, their data, or biological material

Policy information about studies with human participants or human data. See also policy information about sex, gender (identity/presentation), and sexual orientation and race, ethnicity and racism.

Reporting on sex and gender	This study does not involve sex or gender based research.
Reporting on race, ethnicity, or other socially relevant groupings	This study includes an investigation of admixed population's effect on the accuracy of causal gene regulatory network inference. This is purely based on genetic information. No race, ethnicity or other socially relevant groupings were involved.
Population characteristics	This study uses published data. Population characteristics were previously reported.
Recruitment	This study does not involve recruitment.
Ethics oversight	Not applicable.

Note that full information on the approval of the study protocol must also be provided in the manuscript.

Field-specific reporting

Please select the one below that is the best fit for your research. If you are not sure, read the appropriate sections before making your selection.

- [x] Life sciences
- [ ] Behavioural & social sciences
- [ ] Ecological, evolutionary & environmental sciences

For a reference copy of the document with all sections, see nature.com/documents/nr-reporting-summary-flat.pdf

Life sciences study design

All studies must disclose on these points even when the disclosure is negative.

Sample size	This study uses published datasets whose sample sizes were predetermined. The number of datasets was determined by those needed to illustrate the methodological advance.
Data exclusions	We primarily followed the quality control established for high-MOI single-cell CRISPR screen in the Normalisr study [5], with additional criteria for donor-based statistics. Briefly, we removed cells with less than 100 expressed genes or 500 reads, genes expressed in less than 10% of donors, 100 cells, or 2% cells, (cells from) donors with 5 cells or fewer, and SNPs that could not be confidently identified for any donor in the original study.<br><br>The above removal was performed separately for each cell subset (e.g. by cell type according to existing annotations) in published (and possibly already QCed) population-scale scRNA-seq data and iteratively until no gene, cell, SNP, or donor was removed. Then, cell subsets with less than 500 cells, 40 donors, 10,000 SNPs, or 500 genes remaining were excluded from sceQTL mapping and downstream analyses. Here specifically, a gene was defined as “expressed” if it exhibited at least 1 read in a particular cell or any cell from a particular donor.
Replication	Computational scalability evaluation of sceQTL mapping methods were replicated under different gene, SNP, donor, and sample sizes.
Randomization	This study uses published datasets whose randomization and experimental group allocation were predetermined. Different hidden covariate controlling methods were tested and compared: principal components, random effects, and P value correction.
Blinding	This study uses published datasets whose blinding was not possible. Blinded group allocation was not possible because the initial submission was single-authored and was not needed because the analyses were either performed on all group allocations or on those selected objectively e.g. by cell count.

Reporting for specific materials, systems and methods

We require information from authors about some types of materials, experimental systems and methods used in many studies. Here, indicate whether each material, system or method listed is relevant to your study. If you are not sure if a list item applies to your research, read the appropriate section before selecting a response.

Materials & experimental systems

n/a	Involved in the study
☒	☐ Antibodies
☒	☐ Eukaryotic cell lines
☒	☐ Palaeontology and archaeology
☒	☐ Animals and other organisms
☒	☐ Clinical data
☒	☐ Dual use research of concern
☒	☐ Plants

Methods

n/a	Involved in the study
☒	☐ ChIP-seq
☒	☐ Flow cytometry
☒	☐ MRI-based neuroimaging

Plants

Seed stocks	*Report on the source of all seed stocks or other plant material used. If applicable, state the seed stock centre and catalogue number. If plant specimens were collected from the field, describe the collection location, date and sampling procedures.*
Novel plant genotypes	*Describe the methods by which all novel plant genotypes were produced. This includes those generated by transgenic approaches, gene editing, chemical/radiation-based mutagenesis and hybridization. For transgenic lines, describe the transformation method, the number of independent lines analyzed and the generation upon which experiments were performed. For gene-edited lines, describe the editor used, the endogenous sequence targeted for editing, the targeting guide RNA sequence (if applicable) and how the editor was applied.*
Authentication	*Describe any authentication procedures for each seed stock used or novel genotype generated. Describe any experiments used to assess the effect of a mutation and, where applicable, how potential secondary effects (e.g. second site T-DNA insertions, mosiacism, off-target gene editing) were examined.*
